# Host-Mediated Copper Stress Is Not Protective against Streptococcus pneumoniae D39 Infection

**DOI:** 10.1128/spectrum.02495-22

**Published:** 2022-11-22

**Authors:** Stephanie L. Neville, Bliss A. Cunningham, Eve A. Maunders, Aimee Tan, Jacinta A. Watts, Katherine Ganio, Bart A. Eijkelkamp, Victoria G. Pederick, Raquel Gonzalez de Vega, David Clases, Philip A. Doble, Christopher A. McDevitt

**Affiliations:** a Department of Microbiology and Immunology, The Peter Doherty Institute for Infection and Immunity, The University of Melbourne, Melbourne, Victoria, Australia; b Department of Molecular and Biomedical Science, School of Biological Sciences, University of Adelaide, Adelaide, South Australia, Australia; c The Atomic Medicine Initiative, University of Technology, Broadway, Sydney, New South Wales, Australia; d Institute of Chemistry, University of Graz, Graz, Austria; University of Wollongong

**Keywords:** *Streptococcus pneumoniae*, antimicrobial, copper tolerance, glutathione, metal intoxication, murine infection, antimicrobial activity

## Abstract

Metal ions are required by all organisms for the chemical processes that support life. However, in excess they can also exert toxicity within biological systems. During infection, bacterial pathogens such as Streptococcus pneumoniae are exposed to host-imposed metal intoxication, where the toxic properties of metals, such as copper, are exploited to aid in microbial clearance. However, previous studies investigating the antimicrobial efficacy of copper *in vivo* have reported variable findings. Here, we use a highly copper-sensitive strain of S. pneumoniae, lacking both copper efflux and intracellular copper buffering by glutathione, to investigate how copper stress is managed and where it is encountered during infection. We show that this strain exhibits highly dysregulated copper homeostasis, leading to the attenuation of growth and hyperaccumulation of copper *in vitro.* In a murine infection model, whole-tissue copper quantitation and elemental bioimaging of the murine lung revealed that infection with S. pneumoniae resulted in increased copper abundance in specific tissues, with the formation of spatially discrete copper hot spots throughout the lung. While the increased copper was able to reduce the viability of the highly copper-sensitive strain in a pneumonia model, copper levels in professional phagocytes and in a bacteremic model were insufficient to prosecute bacterial clearance. Collectively, this study reveals that host copper is redistributed to sites of infection and can impact bacterial viability in a hypersusceptible strain. However, in wild-type S. pneumoniae, the concerted actions of the copper homeostatic mechanisms are sufficient to facilitate continued viability and virulence of the pathogen.

**IMPORTANCE** Streptococcus pneumoniae (the pneumococcus) is one of the world’s foremost bacterial pathogens. Treatment of both localized and systemic pneumococcal infection is becoming complicated by increasing rates of multidrug resistance globally. Copper is a potent antimicrobial agent used by the mammalian immune system in the defense against bacterial pathogens. However, unlike other bacterial species, this copper stress is unable to prosecute pneumococcal clearance. This study determines how the mammalian host inflicts copper stress on S. pneumoniae and the bacterial copper tolerance mechanisms that contribute to maintenance of viability and virulence *in vitro* and *in vivo*. This work has provided insight into the chemical biology of the host-pneumococcal interaction and identified a potential avenue for novel antimicrobial development.

## INTRODUCTION

Streptococcus pneumoniae (the pneumococcus) is a globally significant pathogen and the leading cause of bacterial pneumonia. Although asymptomatically carried in the nasopharynx of <10% of adults and up to 65% of children (1), S. pneumoniae is also able to disseminate into the lower respiratory tract and the blood, leading to invasive pneumococcal disease (IPD). The incidence of IPD is overrepresented in pediatric and elderly populations, with these population subsets accounting for a majority of the ~1 million pneumococcal-related deaths every year (2, 3). Despite an active vaccine program, rates of multidrug resistance (MDR) in S. pneumoniae are continuing to rise. In 2019 alone ~600,000 deaths were associated with MDR pneumococcal infections, with a further ~150,000 deaths directly attributable to antibiotic resistance (4).

The acquisition of essential nutrients within the mammalian host is required for pneumococcal viability and virulence. Metal ions, such as the *d*-block elements manganese (Mn) and zinc (Zn), are required by all known organisms to support the cellular chemistry of life. The essentiality of nutrient metal ions is targeted by the innate immune response through an array of withholding processes broadly termed nutritional immunity (5–7). Irrespective of the specific withholding mechanism, nutritional immunity serves to deprive invading pathogens of essential elements and abrogate microbial propagation. However, the chemical properties of select metal ions also enable their use as antimicrobials, either directly or indirectly, against invading bacteria. Host-mediated intoxication of microbial pathogens by copper (Cu) or Zn ions has been shown to contribute to infection control (8–13). In studies of bacterial infection, host-mediated metal intoxication has been primarily reported to occur within specific subcellular compartments, such as the phagolysosome of phagocytic cells (11–15), or as a result of tissue-level redistribution of Cu, suggested to be due to the Cu-containing ferroxidase ceruloplasmin (CP) (16, 17). Although multiple lines of evidence implicate Cu as an important antimicrobial metal ion at the host-pathogen interface, studies investigating the impact of abrogating Cu homeostasis on bacterial virulence have reported both variable and contradictory findings. Disruption of bacterial Cu homeostasis has been shown to have impacts ranging from minimal or no perturbation of virulence (18–23) to a significant reduction or attenuation of virulence (15, 24, 25) depending on the bacterial species. Notwithstanding the technical complexity of the different infection models, the apparent lack of an impact on bacterial virulence in some organisms and the variation in study findings highlight that the mechanistic basis for the antimicrobial activity of Cu is not well defined and warrants further investigation.

In S. pneumoniae, prior studies of Cu homeostasis have established that it lacks a dedicated Cu import system and known Cu-dependent metalloenzymes (26). Resistance to exogenous Cu stress is primarily dependent upon the Cu(I)-exporting P_1B_-type ATPase transporter CopA (15, 22, 26) and the Cu(I) metallochaperone CupA (26–28). These two proteins act in concert to tightly regulate cellular Cu abundance (29), and their expression is transcriptionally controlled by the Cu-responsive metalloregulator CopY (27, 29). The lack of an apparent requirement for cellular Cu suggests that the maintenance of the S. pneumoniae Cu efflux machinery, despite host-adaptation genome decay, has been driven by exposure to Cu stress during infection. However, murine infection models of S. pneumoniae Δ*copA* strains have reported distinctly different virulence defects, ranging from no perturbation to complete attenuation of virulence (15, 22). Thus, the antimicrobial role of Cu in the host control of S. pneumoniae infection remains unclear. The variation in virulence defects may also be attributable, at least in part, to the contribution of other stochastic factors, such as the presence of proteins, peptides, and/or low-molecular-weight (LMW) molecules with Cu chaperone/buffering capability, such as γ-l-glutamyl-l-cysteinylglycine (GSH; reduced glutathione). S. pneumoniae does not encode a complete pathway for the biosynthesis of LMW thiols, such as GSH, bacillithiol, mycothiol, or ergothioneine. Instead, the pneumococcus is dependent on acquiring host GSH via the solute-binding protein GshT (30, 31), and possibly the ATP-binding cassette importer MetNP (32). Deletion of *gshT* abrogates pneumococcal GSH accumulation and is associated with a reduction in bacterial virulence in murine infection models and increased *in vitro* susceptibility to oxidative and metal ion stress (30, 31).

In this study, we investigated the roles of Cu efflux and intracellular buffering via GSH in maintaining Cu homeostasis in S. pneumoniae, both *in vitro* and *in vivo.* We report that in the absence of copper efflux (Δ*copA*) and GSH buffering (Δ*gshT*), the double-knockout strain (Δ*copA* Δ*gshT*) was substantially more susceptible to Cu stress *in vitro* than the wild-type or the individual mutant strains. Using a murine model of pneumococcal infection, the Δ*copA* Δ*gshT* strain showed reduced survival *in vivo* relative to the wild-type strain and the Δ*gshT* strain, suggesting that it may be exposed to antimicrobial Cu stress. An analysis of murine tissues for total Cu abundance and spatial distribution of Cu within tissue sections revealed that the concentration of the metal increased in specific niches upon infection. Notably, spatial analyses of Cu distribution within murine lung tissue revealed stochastic, nonuniform enrichment of Cu within spatially distinct regions, providing a potential mechanistic explanation for the apparent variation in virulence studies of S. pneumoniae Δ*copA* strains. Collectively, these data show that host Cu is redistributed in response to pneumococcal infection and has the potential to mediate antimicrobial activity against invading S. pneumoniae. Despite this finding, our work shows that the pneumococcus employs a combination of Cu resistance mechanisms that subvert intoxication and enable systemic dissemination of the pathogen.

## RESULTS

### S. pneumoniae D39 Δ*copA* Δ*gshT* is highly susceptible to copper intoxication.

Copper tolerance in S. pneumoniae relies primarily on the *cop* operon, with auxiliary mechanisms, such as GSH-Cu buffering, also shown to contribute (15, 22, 26, 30). Given the lack of any known Cu uptake pathways in S. pneumoniae, we sought to ascertain the prevalence and conservation of the genes associated with Cu tolerance. An examination of 20,027 publicly available S. pneumoniae clinical isolate genomes revealed that these systems are highly conserved throughout pneumococcal isolates. The genes of the *cop* operon, namely, *copY*, *cupA*, and *copA*, were present in 99.98%, 99.9%, and 99.79% of the genomes analyzed, respectively. Amino acid sequence conservation exceeded 99% pairwise identity for all genes (see Table S1 in the supplemental material). Similarly, an analysis of *gshT* revealed that the gene is present in 99.91% of all clinical isolates, and amino acid sequence conservation exceeded 99% pairwise identity. Thus, these data highlight that the primary and auxiliary Cu tolerance systems of S. pneumoniae have been retained with high fidelity.

**FIG 1:**
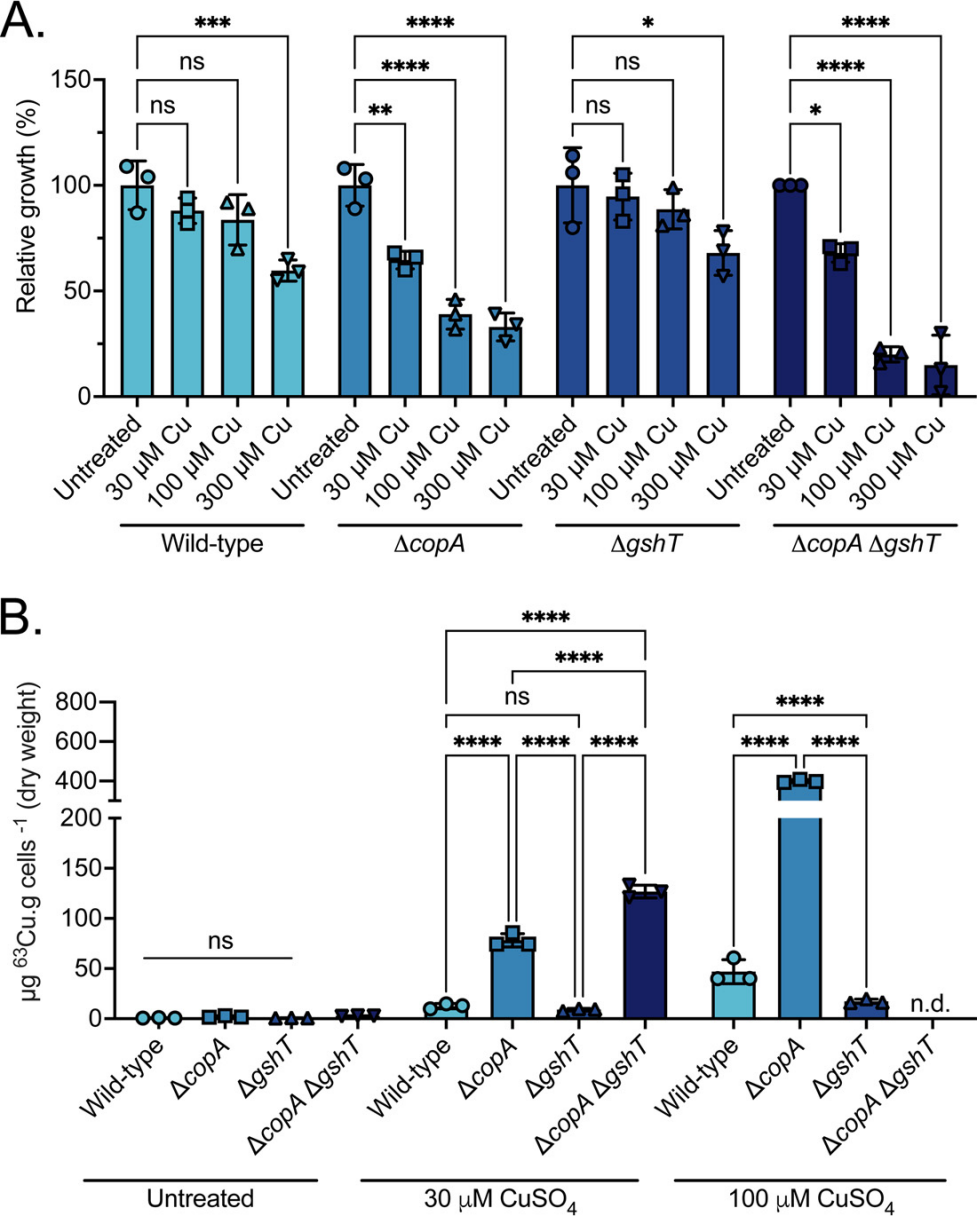
Effect of copper on S. pneumoniae wild-type and mutant strains. (A) Growth phenotypes of S. pneumoniae D39 (wild-type [WT]) and the mutant derivative strains Δ*copA*, Δ*gshT*, and Δ*copA* Δ*gshT* in the presence of CuSO_4_ supplementation (0 μM to 300 μM). Data are presented as % growth relative to untreated (*t* = 240 min) for each strain. Data represent mean (± SEM) optical density at 600 nm (OD_600_) measurements from three independent biological experiments. (B) Whole-cell accumulation of ^63^Cu in S. pneumoniae D39 WT and the mutant derivative strains Δ*copA*, Δ*gshT*, and Δ*copA* Δ*gshT* grown in medium supplemented with 0 μM, 30 μM, and 100 μM CuSO_4_. Data correspond to the mean ± SD of ^63^Cu accumulation (μg ^63^Cu · g cells^−1^ [dry weight]) of 3 independent biological replicates. Statistical significance of the differences was determined by one-way ANOVA with Tukey posttest; *, *P* ≤ 0.05; **, *P* ≤ 0.01; ***, *P* ≤ 0.001; ****, *P* ≤ 0.0001; n.d., not determined; ns, not significant.

Humans are the only known reservoir for S. pneumoniae. Therefore, it logically follows that the conservation of these genes across pneumococcal clinical isolates indicates that they serve a crucial role in preserving viability within humans. Accordingly, we investigated the independent and combined contributions of CopA, the Cu(I)-efflux pathway, and GshT, glutathione import, to S. pneumoniae D39 Cu tolerance. This investigation was performed by constructing the isogenic deletion strains Δ*copA*, Δ*gshT*, and Δ*copA* Δ*gshT* in S. pneumoniae D39 and performing a series of phenotypic assays. Bacterial growth kinetics were investigated in cation-defined medium (CDM) supplemented with increasing CuSO_4_ concentrations (0 to 300 μM). In S. pneumoniae D39 wild-type and Δ*gshT* strains, the minimal level of supplementation required to substantially reduce the relative growth was 300 μM CuSO_4_ (Fig. 1A; see Fig. S1 in the supplemental material). Notably, in the absence of Cu stress, the Δ*gshT* strain achieved a lower final cell density, relative to wild-type, under all conditions tested (Fig. S1). This growth perturbation is likely the result of the alternative cellular roles fulfilled by GSH and is consistent with prior studies (30, 31). The Δ*copA* strain showed a significant reduction in growth in response to CuSO_4_ concentrations of 30 to 300 μM, relative to the untreated (Fig. 1A). Although optical density at 600 nm (OD_600_) measurements showed that the cultures were able to grow, the final cell density was reduced compared to that of the wild-type (Fig. S1). Taken together, these data show that while loss of CopA does impair S. pneumoniae Cu tolerance, resistance is not abrogated. Therefore, we next investigated the contribution of GSH to Cu tolerance using the Δ*copA* Δ*gshT* strain. The growth of this strain was substantially reduced upon exposure to 30 μM CuSO_4_ and severely perturbed at higher concentrations, compared with both the wild-type and Δ*gshT* strains (Fig. 1A, Fig. S1). Collectively, these data show that S. pneumoniae achieves optimal Cu tolerance via the concerted actions of CopA and GshT.

### Copper uptake in S. pneumoniae is an uncoordinated process.

In S. pneumoniae, Cu homeostasis is highly responsive to the extracellular abundance of the metal (15, 22, 26, 27, 33). However, in the absence of a dedicated uptake pathway and an extracellularly responsive Cu sensor-regulator system, such as CusRS (34), Cu is sensed directly within the cytoplasm by the metalloregulator CopY. Consequently, this suggests that Cu accumulation is regulated by efflux (CopA) and buffering (GSH) rather than by uptake. This inference was tested by determining whole-cell Cu accumulation in wild-type S. pneumoniae and mutant derivative strains lacking the primary export pathway and/or the auxiliary buffering pool (Fig. 1B).

Whole-cell accumulation of Cu was negligible in all strains during growth in unsupplemented culture medium, which contained <1 μM of Cu (Fig. 1B). Supplementation with 30 μM CuSO_4_, a subinhibitory concentration, revealed that the Δ*copA* and Δ*copA* Δ*gshT* strains accumulated significantly more Cu than the wild-type or Δ*gshT* strains (Fig. 1B). The accumulation of Cu was also greater than under unsupplemented conditions, which is consistent with CopA serving as the primary Cu efflux pathway (15, 22, 26). Intriguingly, the Δ*copA* Δ*gshT* strain accumulated significantly more Cu than the Δ*copA* strain (126 μg · g cells^−1^ versus 78 μg · g cells^−1^; *P < *0.0001). This result may be attributable, at least in part, to an increased uptake of alternative Cu-buffering molecules, such as sulfur-containing amino acids and peptides, possibly via the activity of the CmbR regulon (35). Further supplementation with CuSO_4_ impaired the growth of the Δ*copA* Δ*gshT* strain preventing the determination of the cellular metal content. In contrast, the Δ*copA* strain was able to maintain growth in 100 μM CuSO_4_, albeit with substantially greater Cu accumulation (Fig. 1B). Given the lack of viability of the Δ*copA* Δ*gshT* strain under this condition, these data support a role for cellular GSH in S. pneumoniae Cu management during acute intoxication. This contribution appears to be crucial when Cu efflux is compromised. In contrast, in the Δ*gshT* strain, 100 μM CuSO_4_ was associated with significantly less Cu accumulation than the Δ*copA* strain and, unexpectedly, the wild-type strain (~17 μg · g cells^−1^ versus 47 μg · g cells^−1^; *P* < 0.0001) (Fig. 1B). This finding may suggest that cellular Cu content is more tightly regulated in the absence of GSH. Supplementation with 300 μM CuSO_4_ prevented the generation of sufficient biomass for an analysis of all strains, except the wild type. S. pneumoniae D39 accumulated 1,031 ± 153 μg · g cells^−1^ (*n* = 3; data not shown) in the presence of 300 μM CuSO_4_, which is nearly 2 orders of magnitude more cellular Cu than upon exposure to 30 μM CuSO_4_ (12 μg · g cells^−1^) (Fig. 1B).

Collectively, these data show that Cu levels increase in all strains upon exposure to CuSO_4_, supporting the inference that uptake is a nonspecific process. Further, these data reinforce the observed primacy of CopA in maintaining cellular Cu homeostasis. This work also highlights the contribution of *gshT*, and by extension GSH, to Cu tolerance. The role of GSH appears to be complementary to CopA, as intracellular Cu abundance is more tightly regulated in its absence (wild-type versus Δ*gshT*), while the toxicity of the metal ion is potentiated in its absence (Δ*copA* versus Δ*copA* Δ*gshT*). Accordingly, we exploited the Cu sensitivity of these strains to investigate Cu utilization by the host during infection.

### Hypersusceptibility to copper does not abrogate survival against host immune cells.

**FIG 2:**
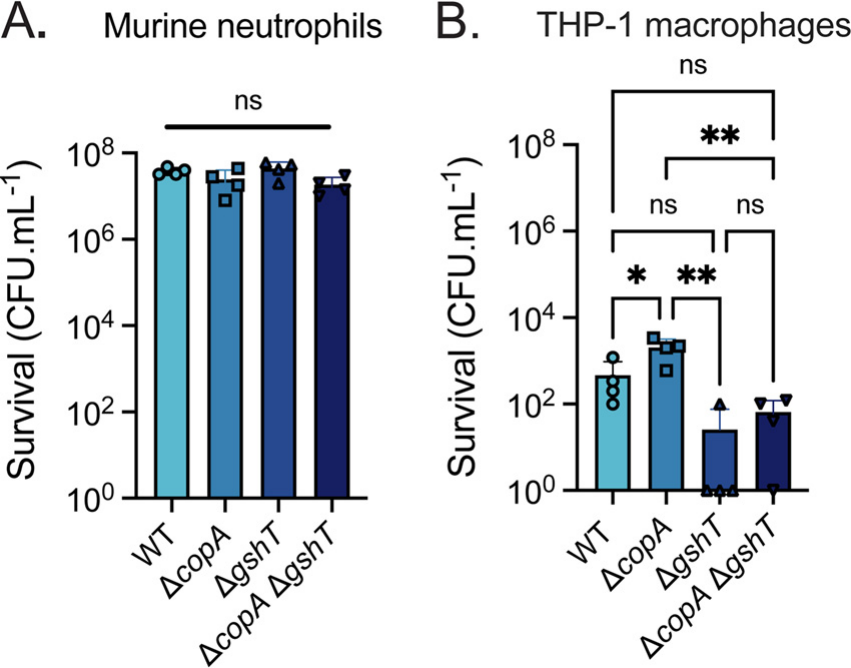
Survival of S. pneumoniae wild-type (WT) and mutant strains following phagocytic cell interactions. Survival of S. pneumoniae WT, Δ*copA*, Δ*gshT*, and Δ*copA* Δ*gshT* incubated with primary murine neutrophils (A) and human-derived THP-1 macrophages (B) to determine resistance to host killing. Data are presented as the total surviving CFU · mL^−1^ of the bacterial strains following incubation with the denoted cell types and represent the mean ± SD of 4 independent biological replicates; *, *P* ≤ 0.05; **, *P* ≤ 0.01; ns, not significantly different; one-way ANOVA with Tukey posttest.

During infection, microbial pathogens are readily captured by macrophages and neutrophils and are exposed to chemical stresses within the phagolysosomal compartment (11, 15, 21, 24, 36). Phagocytic cell Cu levels have been shown to increase in response to infection, with Cu delivered to the phagolysosome via the Cu(I)-transporter ATP7A (24). The antimicrobial efficacy of phagolysosomal Cu has been shown to varying degrees depending on the bacterial species and/or phagocyte lineage (15, 21, 24). Here, the contribution of Cu to S. pneumoniae D39 killing was investigated using primary murine bone-marrow-derived neutrophils to allow a direct comparison with the murine infection model (Fig. 2A). Upon exposure of murine neutrophils to S. pneumoniae strains *ex vivo*, no difference in the relative survival of the wild-type or mutant variants was observed. This finding indicates that, under these experimental conditions, phagolysosomal Cu does not contribute to pneumococci killing within murine neutrophils. To determine if this observation was concordant with human-derived phagocytes, we investigated the contribution of Cu to the control of pneumococci in human THP-1-derived macrophages. Survival of the Δ*gshT* and Δ*copA* Δ*gshT* strains was reduced relative to the wild-type (*P > *0.05; one-way analysis of variance [ANOVA]) and significantly reduced compared with the Δ*copA* strain (*P < *0.01; one-way ANOVA) (Fig. 2B). This result may be due to the other cellular roles fulfilled by GSH, such as resistance to oxidative stress (30, 37), that are implicated in bacterial survival within phagocytes (38). Unexpectedly, the Δ*copA* strain showed a significant, albeit modest, increase in survival relative to the wild-type (*P < *0.05, one-way ANOVA) (Fig. 2B). Thus, a loss of *copA* does not impair pneumococcal survival within THP-1 macrophages. Further, survival of the Δ*copA* Δ*gshT* strain suggests that Cu does not play a significant antimicrobial role against pneumococci in professional phagocytes *in vitro*. It therefore follows that the reduced survival rates of S. pneumoniae Cu homeostasis mutants in prior *in vivo* studies may result from Cu intoxication delivered by an alternative mechanism.

### Host copper levels increase upon S. pneumoniae infection.

**FIG 3:**
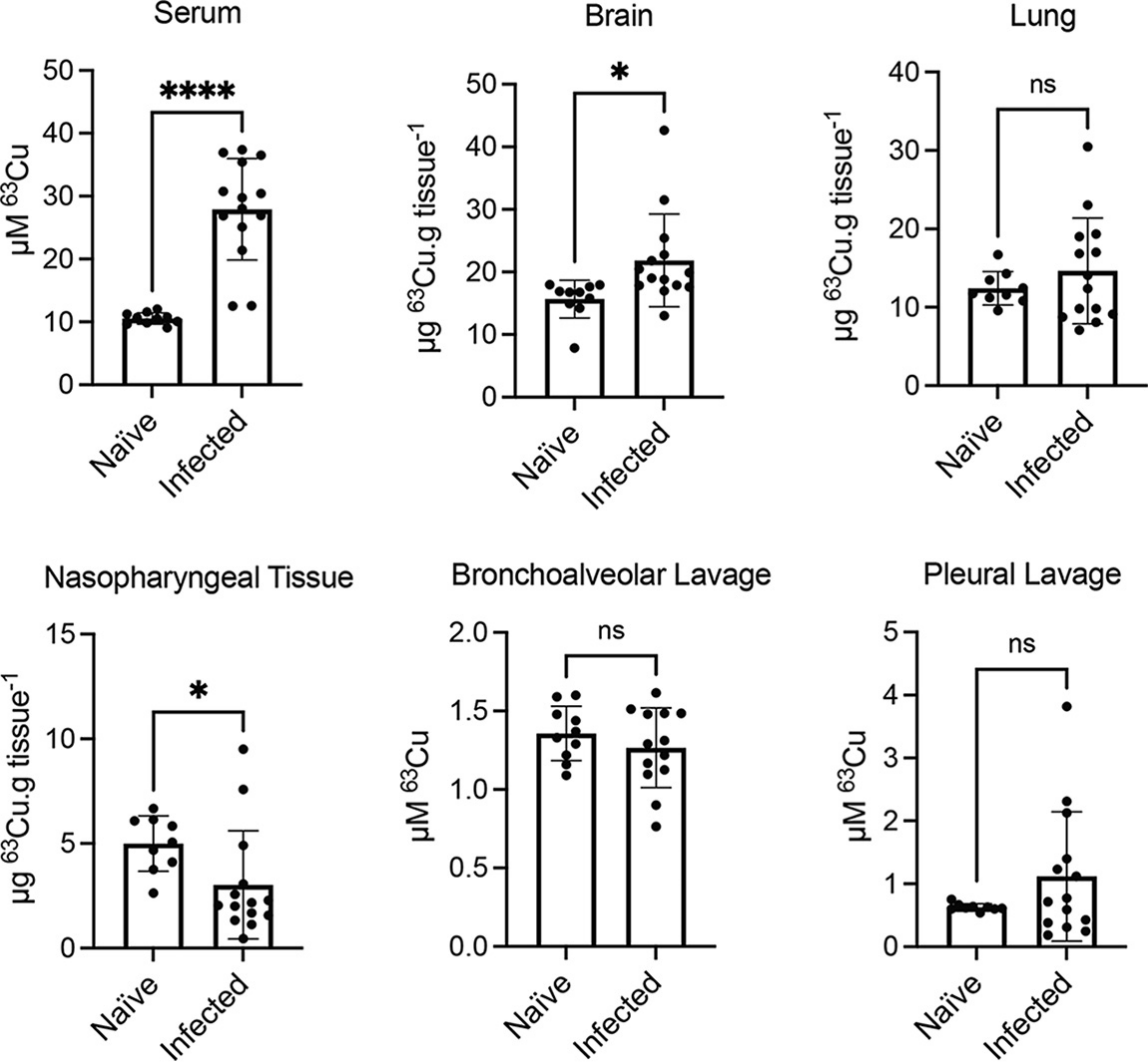
Mouse niche copper concentrations. Total ^63^Cu concentrations in murine tissues determined by ICP-MS. Tissues were harvested from naïve mice (*n *= 10) and mice infected (*n *= 14) with S. pneumoniae D39 wild-type at 36 h post-intranasal infection. Solid tissue ^63^Cu concentration is presented as μg ^63^Cu · g tissue^−1^ (wet weight), and lavage fluid and serum are presented in μM total ^63^Cu. Data represent the mean ± SD, *, *P* ≤ 0.05; ****, *P* ≤ 0.0001; ns, not significantly different; Student’s unpaired *t* test.

The immune system mobilizes Cu during the acute phase response of infection (12, 21, 39). For S. pneumoniae, increases in host tissue Cu abundance have been inferred largely based on pneumococcal transcriptional analyses (22). Here, we directly quantified murine tissue Cu concentrations in the naive state and in response to S. pneumoniae infection. Outbred CD1 Swiss mice were inoculated intranasally with wild-type S. pneumoniae D39, and tissues associated with pneumonia, bacteremia, and meningitis pathologies were harvested at 36 h postinfection (h.p.i.). Concentrations of Cu increased significantly in the serum and the brain (Fig. 3), decreased significantly in the nasopharyngeal tissue, and did not significantly change in the bronchoalveolar lavage (BAL) fluid, pleural lavage (PL) fluid, and lungs in response to infection. Notably, the Cu concentrations within these niches appeared to vary to a greater extent between mice postinfection (Fig. 3). This result may be due to differences in Cu mobilization or variations in infection severity. The representative tissue abundance of Cu followed the order (highest to lowest) of serum (~28 μM ^63^Cu), brain (~22 μg ^63^Cu · g^−1^ tissue), and lung (~15 μg ^63^Cu · g^−1^ tissue). Importantly, these concentrations represent total tissue Cu abundance, with only a proportion likely bioavailable (40). Lavages of other anatomical niches examined had comparatively low levels of total Cu (<2 μM ^63^Cu) (Fig. 3).

Given the substantial increase in serum Cu abundance in response to infection, we investigated the virulence impact associated with the S. pneumoniae Δ*copA*, Δ*gshT*, and Δ*copA* Δ*gshT* strains in a bacteremia infection model.

### Serum copper levels are insufficient for antimicrobial activity.

**FIG 4:**
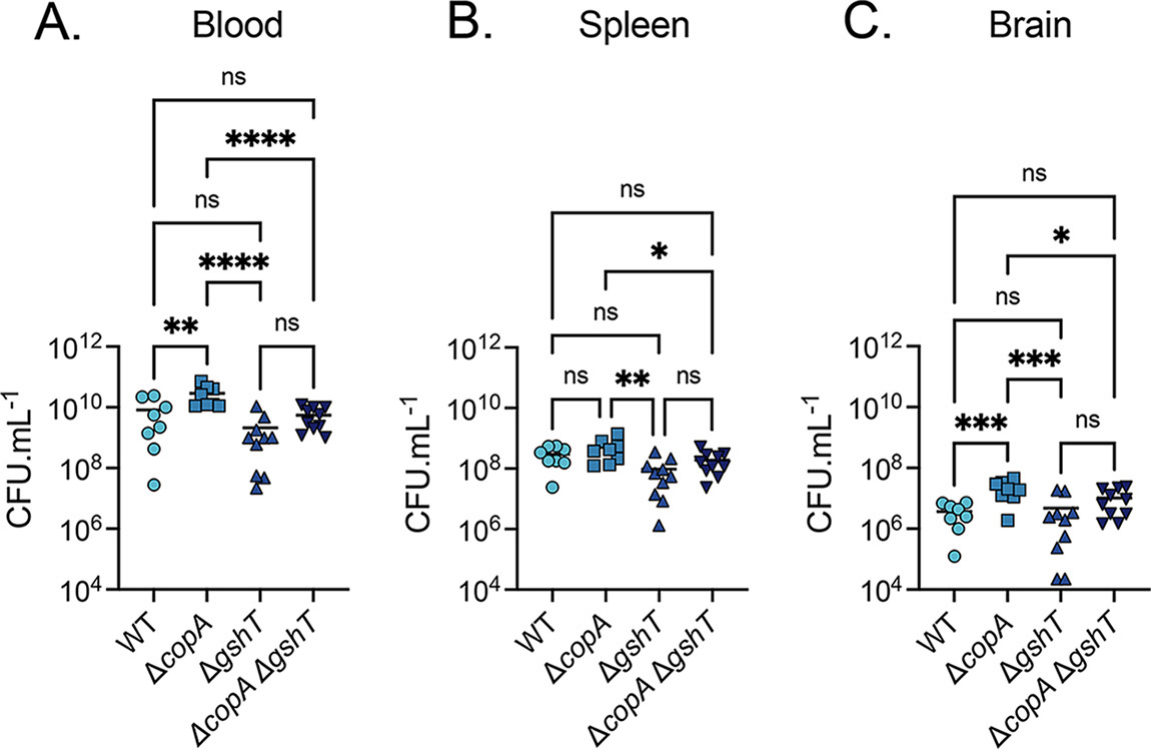
S. pneumoniae intraperitoneal murine challenge. Tissue bacterial load of S. pneumoniae D39 wild-type (WT), Δ*copA*, Δ*gshT*, and Δ*copA* Δ*gshT* at 24-h post-intraperitoneal inoculation of 1 × 10^3^ pneumococci in CD1 Swiss mice. Each datapoint represents an individual animal with the median CFU · mL^−1^ value denoted with a solid line. *, *P* ≤ 0.05; **, *P* ≤ 0.01; ***, *P* ≤ 0.001; ****, *P* ≤ 0.0001; ns, not significantly different; one-way ANOVA.

The antimicrobial potential of host serum Cu was investigated using CD1 Swiss mice infected intraperitoneally (i.p.) with S. pneumoniae D39 wild-type, Δ*copA*, Δ*gshT*, and Δ*copA* Δ*gshT* to induce bacteremia. At 24 h.p.i., severe bacteremia was observed in all mice with an average bacterial burden of ~10^9^ to 10^11^ CFU · mL^−1^ in the blood for all strains (Fig. 4A). These data indicate that all strains were competent for systemic infection. This finding also suggested that, at this time point, the Cu abundance in the blood was either insufficient to overcome the rapid disease progression resulting from i.p. infection or was unavailable to mediate antimicrobial activity. Notably, the bacterial burden of the Δ*copA* strain was greater than that of the wild-type in the blood (*P* = 0.0014) and brain (*P* = 0.0002) and greater than that of Δ*gshT* strain in the blood (*P* < 0.0001), spleen (*P* = 0.0013), and brain (*P* = 0.0002). A similar pattern was observed when comparing the Δ*copA* and Δ*copA* Δ*gshT* strains in the three niches, although the Δ*copA* Δ*gshT* strain had a higher median CFU · mL^−1^ than the Δ*gshT* strain (Fig. 4A to C). The relative increase in the survival of the Δ*copA* and Δ*copA* Δ*gshT* strains, compared with that of the wild-type and Δ*gshT* strains, respectively, suggests that the loss of CopA may confer a selective advantage in the early stages of bacteremia and meningitis. Further, these data show that at 24 h.p.i., serum Cu levels are insufficient to abrogate the viability of the wild-type or mutant strains.

### Copper distribution in the lung is spatially complex.

**FIG 5:**
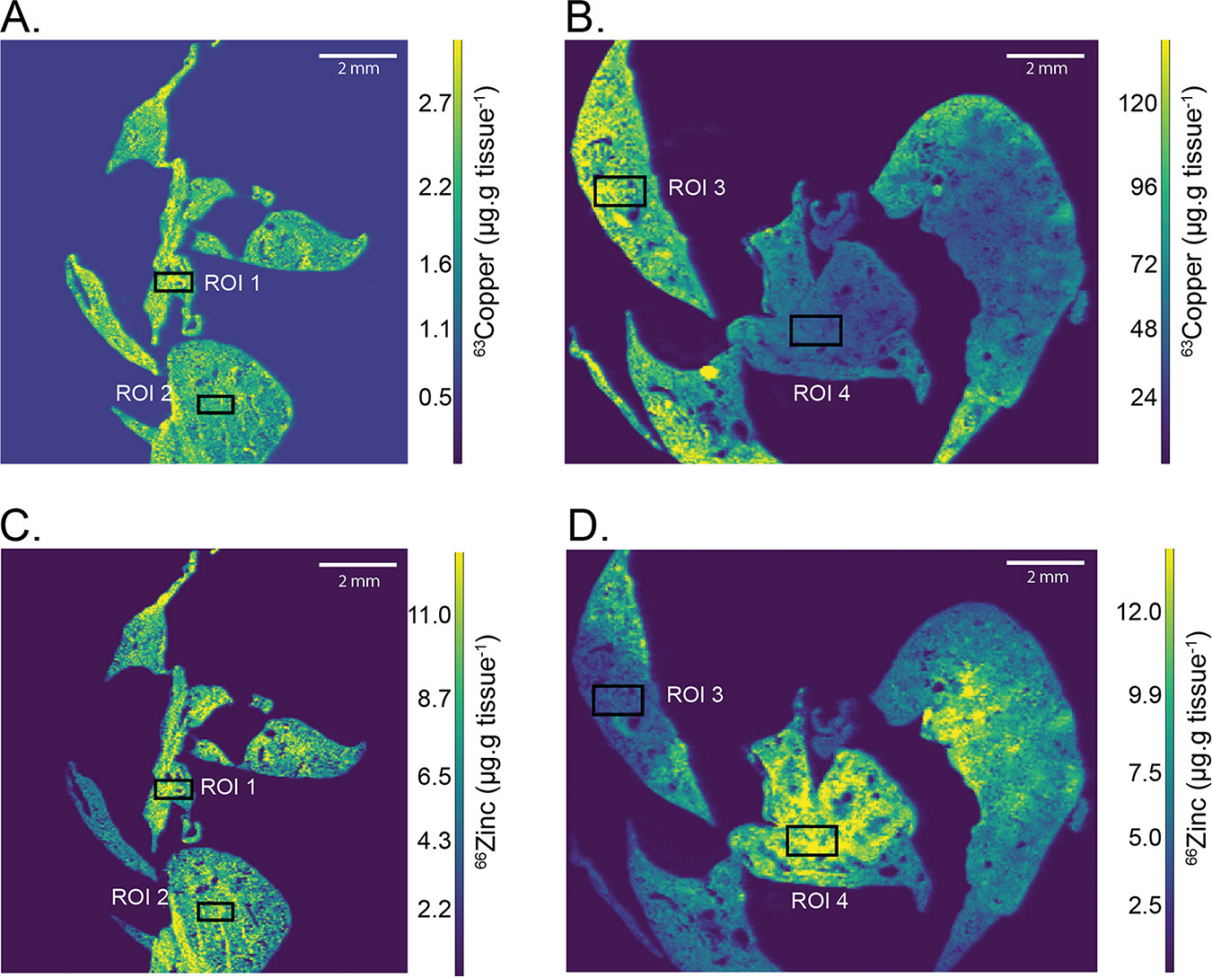
LA-ICP-MS of murine lung tissue. Spatial distribution of ^63^Cu and ^66^Zn throughout naïve and infected murine lung tissue as determined by LA-ICP-MS. Copper concentration (μg ^63^Cu · g tissue^−1^) is shown in A (naïve) and B (D39 infected) presented as a heatmap from low (purple) to high (yellow) ^63^Cu abundance. Zinc concentration (μg ^66^Zn · g tissue^−1^) is shown in C (naïve) and D (D39 infected) presented as a heatmap from low (purple) to high (yellow) ^66^Zn abundance. Regions of interest (ROI) have been denoted by black boxes and are identically placed within the tissue between the ^63^Cu and ^66^Zn analyses. Tissues are representative images from an analysis of 3 mice.

Despite the ability of S. pneumoniae to cause severe sepsis via i.p. infection, the natural route of infection is through the nasopharynx and respiratory tract. During infection, S. pneumoniae disseminates from the upper to the lower respiratory tract with the lungs presenting an anatomical bottleneck (41). Here, specialized alveolar macrophages and other immune factors are recruited to control infection (42, 43). To determine if Cu mobilization into the lungs contributes to host control of pneumococcal infection, elemental bioimaging was performed. This procedure enabled mapping of Cu distribution within murine lung tissue, both before and after infection. Here, mice were inoculated intranasally with wild-type S. pneumoniae, and lung tissue harvested at 36 h.p.i. Elemental bioimaging of naïve lungs revealed relatively even Cu distribution throughout the tissue, with concentrations ranging from ~1 to 4 μg ^63^Cu · g tissue^−1^ in all samples (Fig. 5A) (region of interest [ROI] 1,2). In contrast, at 36 h.p.i., lung tissues displayed a stochastic distribution of Cu, with select regions showing high enrichment ranging from 94 to 126 μg ^63^Cu · g tissue^−1^ (Fig. 5B) (ROI 3). Outside these regions of enrichment, the average lung tissue Cu concentration increased to 20 to 40 μg ^63^Cu · g tissue^−1^ (Fig. 5B) (ROI 4), representing a substantial increase in Cu abundance relative to naïve tissues (see Table S2 in the supplemental material). Taken together, these data show that pneumococcal infection is associated with an increase in murine lung Cu abundance and the emergence of “Cu hot spots.” The spatial complexity of Cu distribution postinfection also highlights the limitations of bulk, whole organ analytical approaches in determining tissue elemental content.

Recent studies have also shown that zinc (Zn) increases in abundance within the murine lung during S. pneumoniae infection (44). Prior observations of Cu and Zn co-occurrence has led to a “brass dagger” postulate, wherein the two elements have been proposed to act synergistically against microbial pathogens (45). Thus, we determined the presence of Zn within murine tissue sections to enable a direct comparison of elemental distribution profiles (Fig. 5C and D). Zinc was relatively evenly dispersed throughout naïve lung tissue, ranging from 6 to 9 μg ^66^Zn · g tissue^−1^ (Fig. 5C) (ROI 1,2). At 36 h.p.i., Zn abundance increased and spatially discrete regions of enrichment (10 to 15 μg ^66^Zn · g tissue^−1^) were observed. Notably, the regions of Zn enrichment occurred only in regions that were absent of elevated Cu (Fig. 5D) (ROI 4). Therefore, these data show that although both Cu and Zn increase in abundance within the murine lung during pneumococcal infection, they occur within spatially discrete regions. Consequently, while S. pneumoniae encounters both Cu and Zn during lung infection, exposure to high concentrations of both metals simultaneously appears to be exceedingly unlikely. Further, prior investigations of elemental remodeling within infected murine lungs showed that the majority of pneumococci were colocalized in regions of increased Zn abundance (44). This information suggested that S. pneumoniae may preferentially colocalize with regions of increased Zn rather than increased Cu. However, the host and/or bacterial factors driving this phenomenon require future elucidation. Based on these observations, we next sought to determine the viability of the Cu-susceptible strains in a pneumonia infection model.

### CopA is not required for virulence in a pneumonia model.

**FIG 6:**
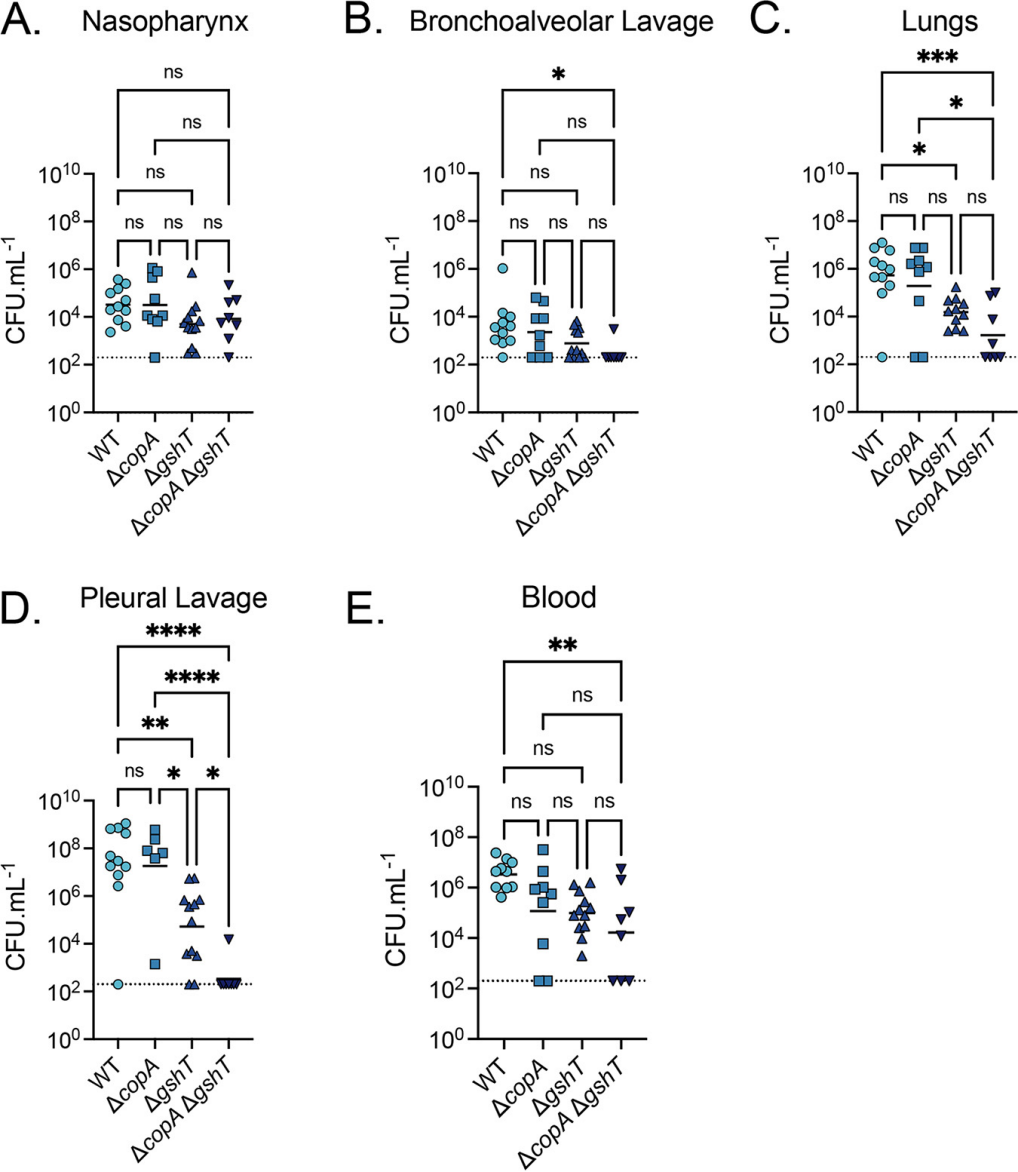
S. pneumoniae intranasal murine challenge. Tissue bacterial load of S. pneumoniae D39 wild-type (WT), Δ*copA*, Δ*gshT*, and Δ*copA* Δ*gshT* at 36-h post-intranasal inoculation of 1 × 10^7^ pneumococci in CD1 Swiss mice. Each datapoint represents an individual animal with the median CFU · mL^−1^ value denoted with a solid line. Limit of detection is shown by a dotted line, with datapoints on the line representing no bacterial recovery from the tissue. *, *P* ≤ 0.05; **, *P* ≤ 0.01; ***, *P* ≤ 0.001; ****, *P* ≤ 0.0001; ns, not significantly different; one-way ANOVA.

Prior studies of S. pneumoniae Δ*copA* mutants in the D39 and TIGR4 backgrounds have reported substantially different virulence defects and bacterial burdens in the lungs and blood (15, 22). These findings render it unclear as to whether Cu mobilized into these niches contributes to host control of infection following the native infection route. To further examine the contribution of host Cu to infection control, we exploited the increased Cu susceptibility of the Δ*copA* Δ*gshT* strain, relative to the wild-type, Δ*copA*, and Δ*gshT* strains. Here, mice were inoculated intranasally with wild-type S. pneumoniae and the mutant strains to mimic the natural route of infection, and tissues were harvested at 36 h.p.i. for bacterial burden enumeration. No significant differences in bacterial burden were observed in the nasopharynx between the wild-type and mutant strains (Fig. 6A). Analyses of the BAL fluid revealed that all strains had a relatively low bacterial burden, ranging from10^2^ to 10^4^ CFU · mL^−1^, with the Δ*copA* Δ*gshT* strain detected only in one mouse (Fig. 6B). While this finding could suggest that the relative abundance of labile Cu was sufficient to exert antimicrobial control, the CFU counts approached the limit of detection for this niche limiting the robustness of conclusions. From the upper respiratory tract, S. pneumoniae invades the lung, followed by dissemination into the pleural cavity and the blood (41, 46). Pneumococcal exposure to antimicrobial Cu stress in the lungs would be predicted to manifest as a reduction in the survival of Δ*copA* Δ*gshT*, relative to the wild-type. We observed that the bacterial burden of the Δ*copA* strain was not significantly different from that of the wild-type in the lungs, pleural cavity, or blood (Fig. 6C to E). This result suggested that the extent of Cu stress encountered was insufficient to compromise the viability of the Δ*copA* strain in the majority of mice. However, despite a lack of statistical significance, the Δ*copA* strain was cleared from a greater number of mice than the wild-type strain, potentially reflecting the stochasticity of Cu distribution. In contrast, although the Δ*gshT* strain showed a significant reduction in bacterial burden in the lungs (*P < *0.05; one-way ANOVA) and pleural lavage fluid (*P < *0.01; one-way ANOVA) compared to the wild-type (Fig. 6C and D) all mice subsequently developed bacteremia (Fig. 6E), illustrating the competence of this strain to cause invasive disease. These data indicate that the inability to acquire host GSH compromises, but does not abrogate, the survival of the Δ*gshT* strain in the lungs, PL fluid, and blood. This phenotype is consistent with previous studies (30) and likely arises due to perturbations in alternative GSH cellular functions during infection.

The Δ*copA* Δ*gshT* strain was detected only in the lungs of four of eight mice at 36 h.p.i. (Fig. 6C). However, five of the eight mice subsequently developed bacteremia, although to a significantly reduced extent compared to that of the wild-type (*P < *0.01; one-way ANOVA) (Fig. 6E). This trend was also reflected in the PL fluid, where only one mouse retained the Δ*copA* Δ*gshT* strain in the pleural cavity, compared with seven of eight mice infected with the wild-type strain (*P < *0.0001; one-way ANOVA). Interestingly, a comparison of the Δ*copA* Δ*gshT* and Δ*gshT* strains revealed that although the recovered bacterial load was not significantly different (with the exception of the PL fluid), the double mutant was cleared more readily by the host in all niches analyzed. This finding suggests that the virulence defect imparted by the loss of *gshT* alone can be exacerbated by the subsequent loss of *copA*. The most likely explanation is that pneumococci within these niches are exposed to an increased abundance of Cu, which mediates a more pronounced impact on the Δ*copA* Δ*gshT* strain. Taken together, these data indicate that, although there is the potential for an antimicrobial role of Cu during infection, the wild-type strain is capable of mitigating this stress with the concerted action of the high-affinity Cu efflux pathway and the intracellular buffering capacity of GSH.

## DISCUSSION

The role of Cu in bacterial infection of mammalian hosts remains poorly defined. Although it is well established that Cu levels increase during infection, whether the metal ion can mediate direct antimicrobial activity at infection sites is unclear. This uncertainty is reflected in numerous studies to date, which have shown that the disruption of bacterial Cu homeostasis can have highly varied impacts, ranging from minimal or no perturbation of virulence, in Listeria monocytogenes (18), Mycobacterium tuberculosis (19), Salmonella enterica serovar Typhimurium (20), Streptococcus pyogenes (21), S. pneumoniae (22), and Vibrio cholerae (23), to a significant reduction or attenuation of virulence in Escherichia coli (24), *S.* Typhimurium (25), and S. pneumoniae (15). While these differences may be reflective of the chosen infection model, it is also possible that bacterial pathogens with less dynamic Cu tolerance systems are more susceptible to sites of increased Cu during infection. Consistent with other studies (22), this work showed that Cu concentrations increase within tissues infected by S. pneumoniae. Elemental bioimaging of murine lungs showed that in response to pneumococcal infection, Cu concentrations increased by up to 120-fold within spatially discrete regions. Regions enriched for Cu were spatially distinct from regions elevated in Zn abundance. Thus, the brass dagger hypothesis for the control of microbial pathogens (i.e., colocalization of Cu and Zn at the site of infection) does not appear to apply within the context of the lung. Furthermore, the presence of spatially discrete regions for the two metal ions suggests a coordinated process of metal intoxication within the lung, although further studies, such as spatial transcriptomics, would be required to assess the veracity of this speculation.

Prior analyses of murine lungs infected with S. pneumoniae showed that the bacteria were colocalized with regions of increased Zn concentration at 36 h.p.i (44). This finding may be a result of the host immune response preferentially delivering the Zn to the site of bacterial infection within the lung, or alternatively, it is possible that the Cu hot spots represent less favorable sites for bacterial survival and have become devoid of their presence. The complex spatial distribution of Cu during bacterial infection may also provide a plausible explanation for the variations in lung infection observed in the murine challenge experiments. Spatial differences in the entry of antimicrobial Cu into the lung may have resulted in some of the strains experiencing a greater level of stress within specific hot spots, resulting in elimination from the lung. This observation is supported by the presence of the Δ*copA* Δ*gshT* strain in the blood, although it had been eliminated from the lungs in the majority of mice. These data provide a basis to address the confounding reports on the role of Cu at the host-pathogen interface for respiratory pathogens. For S. pneumoniae, the complexity of host Cu utilization is highlighted by our analysis of the virulence impact of the Δ*copA* deletion in the serotype 2 D39 strain, presented here and consistent with another prior report in D39 (22). However, these findings contrast starkly as having a substantially modest effect compared with a report on the serotype 4 TIGR4 Δ*copA* strain (15). It is important to note that other confounding factors, such as the use of different mouse strains and/or potential differences in handling of Cu stress between different S. pneumoniae serotypes, may also be underlying factors. Irrespective, further application of elemental bioimaging to map the dynamics of murine lung infection over time and across serotypes would reveal the role of antimicrobial metals, such as Cu and Zn, and address the contribution of spatial complexity to current models.

Elevation of Cu levels within infection sites is attributed predominately to the infiltration of the ferroxidase ceruloplasmin (CP) during the acute-phase response (12, 16, 39, 47). However, biochemical studies have found that the Cu ions are tightly integrated into CP during biosynthesis and are unlikely to exchange with Cu pools during systemic circulation (16, 48, 49). Consequently, although the ferroxidase has been implicated as serving an antimicrobial role (12), direct interaction of the metalloenzyme or its bound metal with invading bacteria remains to be established. Ceruloplasmin-induced Cu stress has been reported for uropathogenic E. coli in human urine *in vitro* (12), but the accessibility of CP-bound Cu to invading pathogens *in vivo* is currently unknown. In contrast, exchangeable sources of Cu, such as albumin, transcuprein, and amino acid-Cu complexes (47, 50) may provide a source of Cu that can interact with invading microbes (48). However, their relative abundance and contribution to Cu intoxication of invading pathogens is essentially unknown. It is also possible that the Cu is delivered via the infiltration of phagocytic cells to sites of infection with Cu loading occurring prior to entry. Irrespective, in a pneumonia infection model, Cu appears to be a component of the host immune response, although it is insufficient to prosecute pneumococcal clearance. In a bacteremic model, the enhanced survival of the Δ*copA* strain in the blood and the brain may suggest that host Cu does not serve an efficacious antimicrobial role against invasive pneumococcal disease. However, this finding is more likely a result of the infection progressing too rapidly (within 24 h), as previous studies of microbial infections have found that Cu levels in the serum peak at 48 to 72 h.p.i. (21, 39). For this reason, S. pneumoniae infection models which are able to progress >48 h.p.i. may yield different results with respect to the survival and virulence of a Δ*copA* strain.

Although the molecular mechanisms of Cu toxicity are still incompletely understood, the importance of a thiol buffering pool to mitigate Cu stress has been demonstrated in numerous organisms (21, 51, 52). Recent studies in S. pyogenes have shown that the GSH buffering pool facilitates Cu tolerance when the primary resistance mechanisms (Cu sensing and efflux) are overwhelmed (21). Unchaperoned Cu has been shown to opportunistically interact with cellular proteins leading to mismetallation (53, 54), inactivation of enzymes (21, 55), or protein aggregation (51, 56). However, maintenance of Cu in the cuprous (+1) state is also reliant on the redox properties of molecules, such as GSH, to sequester and subsequently reduce cupric (+2) ions (51). In the absence of GSH, the cellular environment may become less reducing, possibly leading to an increased abundance of Cu(II). As a borderline hard acid, Cu(II) exhibits greater affinity for nitrogen- and oxygen-coordinating ligands (57) which, when coupled with the highly competitive nature of the Cu(II) ion in biological complexes (58), is likely to greatly increase the incidence of Cu(II) ions interacting with cytoplasmic proteins and perturbing cellular physiology (53–55). As S. pneumoniae does not appear to possess an alternative thiol pool, these mechanisms provide possible explanations for the severely heightened Cu sensitivity of the Δ*copA* Δ*gshT* strain. The lack of an alternative thiol pool may also explain the growth perturbation of the Δ*gshT* strain in the absence of Cu stress *in vitro*, which is likely the result of the alternative cellular functions fulfilled by GSH. These functions include the buffering of other metal ions, oxidative stress management, and cellular redox homeostasis (30, 37, 59, 60). Interestingly, the reduced cellular density of Δ*gshT in vitro* does not appear to hinder the replication or invasiveness of this strain in the bacteremia model *in vivo*. After 24 h of infection, the Δ*gshT* strain was recovered from murine tissues in approximately equivalent numbers to the wild-type strain and achieved comparable dissemination to the brain. This result, together with the reduced survival of the Δ*gshT* strain in the THP-1 survival assay, suggests that the virulence defect seen in the pneumonia model is likely a result of enhanced host clearance in the analyzed niches rather than reduced growth. The greater elimination of the Δ*copA* Δ*gshT* strain, compared with that of the Δ*gshT* strain alone, suggests that Cu stress is indeed further limiting the viability of the double mutant. However, additional analyses, such as a spatiotemporal analysis of Cu stress in a GSH uptake-competent strain, would be required to assess the veracity of this inference.

Collectively, this study provides insight into the complexity of Cu mobilization in host tissues and investigates the mechanisms used by S. pneumoniae to resist Cu stress during infection. However, this work also illustrates that, despite host mobilization of Cu, the increased abundance of the metal is insufficient to contribute to control of wild-type S. pneumoniae D39 infection. The hypersusceptibility of the Δ*copA* Δ*gshT* strain to Cu stress suggests that therapeutic enhancement of host-mediated Cu intoxication against S. pneumoniae may have potential as an antimicrobial strategy.

## MATERIALS AND METHODS

### Growth of S. pneumoniae D39.

S. pneumoniae was grown routinely in cation-defined media (CDM), which corresponded to C+Y media without supplementation of transition metals (61). The base transition metal concentration of the medium was determined by inductively coupled plasma mass spectrometry (ICP-MS) on an Agilent 8900 triple quadrupole ICP-MS (Adelaide Microscopy, University of Adelaide) instrument as described previously (31, 62). Copper concentration of the CDM was <1 μM when unsupplemented. Where noted, CDM was supplemented with 30 μM, 100 μM and 300 μM CuSO_4_. All growth experiments were conducted in CDM supplemented with 1 μM MnSO_4_. All chemicals used in this study were purchased from Merck unless otherwise specified. Cultures of S. pneumoniae D39 were routinely prepared from overnight growth on blood agar plates (39 g · L^−1^ Columbia blood agar base [Oxoid] and 5% [vol/vol] defibrinated horse blood), resuspended, and inoculated into CDM to an optical density at 600 nm (OD_600_) of 0.05 (63). The culture was incubated at 37°C + 5% CO_2_ and grown to an OD_600_ of 0.3. Growth kinetic assays were conducted in a 96-well plate format using a CLARIOstar spectrophotometer (BMG Labtech). S. pneumoniae was inoculated in a final volume of 200 μL CDM (± supplementation where indicated) to a starting OD_600_ of 0.01 in a clear 96-well plate (Greiner). Plates were incubated at 37°C + 5% CO_2_ for >16 h with readings taken every 30 min (64). Growth assay data were analyzed using GraphPad Prism v9. Data represent the mean (± SD) of three independent biological replicates.

### S. pneumoniae mutant strain generation.

The S. pneumoniae Δ*copA* and Δ*copA* Δ*gshT* mutant strains (see Table S3 in the supplemental material) were constructed using the Janus cassette (65) (see Fig. S2 in the supplemental material). Briefly, the *rpsL* gene of S. pneumoniae D39 wild-type or Δ*gshT* was replaced with a streptomycin-resistant allele (D39 *rpsl*^+^) to allow counter selection. The upstream and downstream flanking regions of *copA* (spd_0635) were then amplified using primers (see Table S4 in the supplemental material) with complementarity to the Janus cassette and were joined to the Janus cassette by overlap extension PCR. These linear fragments were used to replace *copA* in the S. pneumoniae D39 wild-type or Δ*gshT* chromosome by homologous recombination. The Janus cassette, containing a kanamycin resistance gene, was used to select successful Δ*copA* transformants. All mutants were sequence confirmed (2 kb up- and downstream) prior to use. Antibiotic resistance cassettes were not removed prior to subsequent analyses. Growth media were supplemented with the following antibiotics (Merck) where appropriate: streptomycin (150 μg · mL^−1^), gentamicin (5 μg · mL^−1^), or kanamycin (200 μg · mL^−1^).

### Bioinformatic analyses of gene conservation.

A database of 20,027 publicly available pneumococcal genome sequences (66) were screened for selected copper homeostasis genes using the BLASTN screening tool, Screen Assembly (v1.2.7) (67), applying cutoffs of 80% identity and 80% reference length. To reduce a false-negative rate due to contig breaks and low homology, gene absences were further validated by screening 3 segments (102 to 600 bp) of each target gene (*gshT*, *copY*, *cupA*, and *copA*). The presence of the target gene was determined by the number of fragment hits (i.e., Present = 3, Partial = 2, Ambiguous = 1, Absent = 0). Details of screened fragments can be found in Table S1. Translated full-length protein sequences were used for variation analysis by MAFFT alignment (v7.489) (68) via the Galaxy Web platform (69).

### Whole-cell copper analysis.

Copper analysis of S. pneumoniae D39 and the mutant variants was performed in CDM with or without CuSO_4_ supplementation as described above. Bacterial cells were grown in 45 mL cultures and harvested at an OD_600_ of ~0.3. Cells were pelleted and then washed twice with phosphate-buffered saline (PBS) + 5 mM EDTA and washed twice with PBS. Cells were again pelleted, the supernatant was removed, and the pellet was desiccated overnight on a heat block at 96°C. Cellular copper was solubilized by treatment with 1 mL of 35% (vol/vol) nitric acid (HNO_3_; Suprapur, Merck) at 96°C for 1 h. Copper content was analyzed on an Agilent 8900 triple quadrupole ICP-MS instrument as described previously (8, 31). Data represent the mean (± SD) of three independent biological replicates.

### Murine tissue total copper determination.

For total tissue copper quantitation, 5- to 6-week-old CD1 Swiss mice were anesthetized with xylazine-ketamine and inoculated intranasally with 1 × 10^7^ S. pneumoniae D39 wild-type. At 36 h postinfection (h.p.i.), infected mice (*n* = 14) and naïve mice (*n* = 10) were euthanized by xylazine-ketamine overdose and anatomically relevant niches were harvested. This time point was selected for all intranasal inoculation murine infection studies as it allowed sufficient time for the innate immune response-mediated redistribution of copper, while minimizing the discomfort of the animals. Blood was collected by syringe from the posterior vena cava. The pleural cavity was lavaged through the diaphragm, and the bronchoalveolar lavage was conducted through the trachea, both with 1 mL sterile PBS. Pulmonary vasculature was perfused by infusion of sterile PBS through the heart and lungs prior to excision. Lastly, the nasopharynx (nasopharyngeal tissue) and brain were excised. Solid tissues were homogenized (Precellys homogenizer). For metal ion determination by ICP-MS, the homogenized tissues were desiccated, weighed, and boiled in 65% (vol/vol) HNO_3_ for 30 min, followed by the addition of H_2_O_2_ to a final concentration of 10% (vol/vol), and they were heated at 70°C for 15 min. The samples were then diluted 1:20 for ICP-MS analysis. The liquid niches were diluted into 35% (vol/vol) HNO_3_ and boiled for 30 min prior to the removal of debris by centrifugation. Samples were diluted to a final concentration of 3.5% (vol/vol) HNO_3_. Metal ion detection by ICP-MS was performed on an Agilent 8900 triple quadrupole ICP-MS instrument (Bio21 Institute, University of Melbourne).

### Murine tissue preparation for LA-ICP-MS.

For LA-ICP-MS analysis, murine lung tissue was harvested from 5- to 6-week-old CD1 Swiss mice inoculated intranasally with 1 × 10^7^ S. pneumoniae D39 wild-type, either naïve (*n* = 3) or 36 h.p.i. (*n* = 3). Murine lungs were perfused with ~10 mL sterile PBS prior to excision. Lung tissues were immediately frozen, using liquid nitrogen and isopentane, to conserve the elemental distribution and content. Lung tissues were sectioned (30 μm) by the Melbourne Histology Platform on a cryotome (Leica CM1860 cryostat) at −20°C, mounted on microscope glass slides (SuperFrost Plus), and then stored at −80°C. Tissues sections were thawed at room temperature immediately prior to analysis.

### Elemental bioimaging.

LA-ICP-MS experiments were performed with a CETAC LSX-213 G2+ laser ablation system (Teledyne CETAC Technologies, USA) and coupled to a Thermo iCAP RQ ICP-MS instrument (Thermo Fisher). Helium was used as the carrier gas (99.999% purity). The LA-ICP-MS system was tuned for maximum sensitivity prior to each experiment using the certified reference material NIST 612 “Trace Elements in Glass.” The ICP-MS instrument was operated in standard mode and tuned to minimize the formation of oxides by monitoring the oxide ratio (^232^Th^16^O^+^/^232^Th^+^, *m/z* 248/232 < 0.3%). The laser beam spot size was 30 μm, and the scan speed was 120 μm · s^−1^. Images were created and quantified using the imaging software MassImager 3.49 (University of Muenster, Germany). For quantification, gelatin-based standards were prepared according to a protocol described by Westerhausen et al. (70). Briefly, Cu and Zn were spiked into heated (liquified) gelatin, mixed and filled into molds, solidified, and dried. Aliquots of the liquid gelatin were dried, dissolved in HNO_3_, and diluted and quantified (triplicate analysis) using ICP-MS. Calibration curves were constructed by plotting the signal intensity of ^66^Zn^+^ and ^63^Cu^+^ obtained by LA-ICP-MS against determined Cu and Zn levels which ranged between 1 and 25 μg · g^−1^. The correlation coefficient as a measure of linearity was greater than 0.999 for both calibrations. Using the obtained linear regressions, each data point (voxel) recorded by LA-ICP-MS was converted into concentrations.

### Murine infection models.

For the pneumonia infection model, 5- to 6-week-old outbred CD1 Swiss mice were anesthetized and inoculated intranasally with 1 × 10^7^ S. pneumoniae D39 wild-type, Δ*copA*, Δ*gshT*, or Δ*copA* Δ*gshT* in 30 μL. The challenge dose was confirmed retrospectively by serial dilution and plating onto blood agar. Mice were monitored twice daily for weight loss and signs of illness. At 36 h.p.i., mice were euthanized and niches were harvested as detailed above for CFU · mL^−1^ determination by plating on blood plates supplemented with 5 μg · mL^−1^ gentamicin to reduce non-pneumococcal recovery from nonsterile niches.

For the bacteremia infection model, 5- to 6-week-old outbred CD1 Swiss mice were anesthetized and inoculated with 1 × 10^3^ S. pneumoniae D39 wild-type, Δ*copA*, Δ*gshT*, or Δ*copA* Δ*gshT* in 100 μL directly into the peritoneal cavity. The challenge dose was confirmed retrospectively by serial dilution and plating onto blood agar. Mice were euthanized 24 h.p.i. to compensate for the faster disease progression, and anatomical niches associated with bacteremia and meningitis were harvested for CFU · mL^−1^ determination by dilution plating.

### Macrophage killing assays.

THP-1 monocytes (ATCC TIB-202) were grown under atmospheric control at 37°C + 5% CO_2_ in complete RPMI medium (RPMI with phenol red [Gibco], supplemented with 10% fetal bovine serum, 10 mM HEPES, 30 μg · mL^−1^ penicillin, and 50 μg · mL^−1^ streptomycin). For killing assays, monocytes were seeded into 24-well plates at a cell density of 2 × 10^5^ cells · well^−1^ in 500 μL and were differentiated with 100 ng · mL^−1^ phorbol 12-myristate 13-acetate (PMA; Merck) for 72 h at 37°C + 5% CO_2_ (63). Attached differentiated THP-1 cells (macrophages) were washed in complete RPMI and incubated with complete RPMI without added PMA to allow resting for a further 24 h. Immediately prior to the addition of S. pneumoniae, RPMI was aspirated from wells and replaced with Hank’s balanced salt solution (HBSS; Thermo Fisher Scientific). Wild-type S. pneumoniae D39 and the mutant strains were grown overnight on blood agar plates at 37°C + 5% CO_2_ and subsequently inoculated into CDM to an OD_600_ of 0.05. The cultures were grown to an OD_600_ of 0.3, after which the cells were washed and resuspended in HBSS, and CFU counts were determined by plating onto blood agar. The macrophages and S. pneumoniae cells were coincubated at a ratio of 1:10 for 30 min. The macrophages were then washed, and extracellular bacteria were killed by incubation with 200 μg · mL^−1^ gentamicin and 10 μg · mL^−1^ penicillin for 30 min. The macrophages were washed in HBSS without antibiotic and incubated for a further 30 min. Bacterial enumeration was performed by lysing the macrophages with 0.0625% (wt/vol) Triton X-100 and subsequent plating onto blood agar to determine survival (CFU · mL^−1^). Data represent the mean (± SD) of four independent biological replicates.

### Neutrophil isolation and killing assay.

Primary murine neutrophils were harvested from the bone marrow of naïve, 10-week-old BALB/c mice essentially as reported in reference 71. Briefly, murine femurs and tibias were isolated and excess tissue removed. The bones were sterilized in ethanol for 5 min prior to being washed in PBS thrice. The bone marrow was flushed using PBS + 2% (vol/vol) FCS and passed through a 70 μm cell strainer. Histopaque purification was then used to isolate the neutrophils. Briefly, histopaque-1077 was gently added to histopaque-1119 and cell suspension layered on top. Samples were spun for 700 × *g* for 30 min at room temperature, with no brake. The lower layer was then removed and centrifuged for 400 × *g* for 10 min. Cells were washed once with PBS. The purity of neutrophils was confirmed by flow cytometry prior to analysis and counted on a hemocytometer.

For the S. pneumoniae killing assays, the neutrophils and S. pneumoniae strains were resuspended in HBSS and incubated together in a 96-well plate at a multiplicity of infection (MOI) of 10:1 for 60 min at 37°C + 5% CO_2_. After incubation, the wells were spiked with 0.0625% Triton X-100 and mixed via pipetting. All wells were then serially diluted in sterile PBS and plated onto blood agar for CFU · mL^−1^ determination. Data represent the mean (± SD) of four independent biological replicates.

### Animal ethics.

All animal experiments conducted at the University of Melbourne were approved by the University of Melbourne Animal Ethics Committee (project approval number 1814663) and were performed in strict adherence to guidelines dictated by the Australian Code of Practice for the Care and Use of Animals for Scientific Purposes. Mice were anesthetized by intraperitoneal injection of 5 mg · kg^−1^ xylazine and 75 mg · kg^−1^ ketamine and euthanized by xylazine-ketamine overdose (50 mg · kg^−1^/500 mg · kg^−1^, respectively).

All animal experiments conducted at the University of Adelaide were approved by the University of Adelaide Animal Ethics Committee (Animal Welfare Assurance number A5491-01; project approval number S-2013-053) and were performed in strict adherence to guidelines dictated by the Australian Code of Practice for the Care and Use of Animals for Scientific Purposes. Mice were anesthetized by intraperitoneal injection of pentobarbital sodium (Nembutal; Rhone-Merieux) at a dose of 66 μg · g body weight^−1^. Mice were euthanized by CO_2_ asphyxiation.

### Statistical analysis.

Statistical analyses were performed with the Prism software (GraphPad Prism v9). Grouped data were analyzed by one-way ANOVA followed by multiple comparisons (Tukey posttest). Nongrouped analyses were performed using the Student’s unpaired *t* test. Statistical significance was computed at a *P* value of ≤0.5, as follows: *, *P* ≤ 0.05; **, *P* ≤ 0.01; ***, *P* ≤ 0.001; and ****, *P* ≤ 0.0001. The numbers of animals and replicates for each experiment are indicated in the figure legends.
