# Supplementary material for: Host-Mediated Copper Stress Is Not Protective against Streptococcus pneumoniae D39 Infection
Source: Microbiol Spectr. 2022 Nov 22;10(6):e02495-22. doi: 10.1128/spectrum.02495-22 (PMC9769658; doi:10.1128/spectrum.02495-22)
Supplement: Supplemental file 1 — Tables S1 to S4 and Fig. S1 and S2. Download spectrum.02495-22-s0001.pdf, PDF file, 0.3 MB [file spectrum.02495-22-s0001.pdf]

SUPPLEMENTARY MATERIAL

Supplementary Table 1: Genomic screening of copper tolerance genes in *S. pneumoniae* clinical isolates

		gshT	*copY*	*cupA*	*copA*
**Counts**	**Present***	19976	19972	19952	19968
	**Partial***	29	45	56	15
	**Ambiguous***	3	6	16	2
	**Sum hits**	20008	20023	20024	19985
	**Premature stop***	18	3	2	41
	**Absent***	1	1	1	1
**Ratios**	**Present***	0.9975	0.9973	0.9963	0.9971
	**Partial***	0.0014	0.0022	0.0028	0.0007
	**Ambiguous***	0.0001	0.0003	0.0008	0.0001
	**Total hits**	0.9991	0.9998	0.9999	0.9979
	**Premature stop***	0.0009	0.0001	0.0001	0.0020
	**Absent***	0.0000	0.0000	0.0000	0.0000
	**Total**	1.0000	1.0000	1.0000	1.0000
**Variation**	**Nucleotide pairwise identity (%)^**	98.6	98.5	99.1	99.2
	**Unique amino acid variants^**	372	122	109	764
	**Amino acid pairwise identity (%)^**	99.4	99.2	99.0	99.2

*Gene status (present, absent, partial, ambiguous) as determined by full length and fragment screening, with status assigned as per the key below

^Variation data calculated on full length data - excludes partial and ambiguous hits

***KEY**	*gshT*	*copY*	*cupA*	*copA*
**Gene length (bp)**	831	396	372	2244
**Gene fragments screened**	3	3	3	3
**Fragment size (bp)**	252	102	102	600
***Definition**	**Fragments present in genome**			
***Present**	3	3	3	3
***Partial**	2	2	2	2
***Ambiguous**	1	1	1	1
***Absent**	0	0	0	0

Supplementary Table 2: LA-ICP-MS murine lung metal concentrations

	Naïve						Infected					
	Mouse 1		Mouse 2		Mouse 3		Mouse 1		Mouse 2		Mouse 3	
	ROI 1	ROI 2	ROI 1	ROI 2	ROI 1	ROI 2	ROI 3	ROI 4	ROI 3	ROI 4	ROI 3	ROI 4
Cu (µg/g tissue)	2.7701	4.2583	2.5610	2.0766	2.2293	1.3248	94.5257	21.4161	126.4984	36.3910	100.5147	37.9533
Zn (µg/g tissue)	7.8245	9.3793	8.9123	8.8953	6.5403	4.2228	6.8882	15.5144	3.4773	10.6307	3.4637	12.2605
Cu/Zn ratio	0.3540	0.4540	0.2874	0.2334	0.3409	0.3137	13.7228	1.3804	36.3779	3.4232	29.0191	3.0956

* The ROIs for both the naïve and infected mice were selected as per Fig. 5, illustrating the high and low Cu regions for the infected mice. Reported values denote the metal concentration averaged across the ROI.

Supplementary Table 3: Strains used in the study

**Strain**	**Description**	**Source**
S. pneumoniae serotype 2 D39	NCTC7466	National Collection of Type Cultures
S. pneumoniae D39 Δ*copA*	Spd_0635 replaced with kanamycin resistance cassette	This study
S. pneumoniae D39 Δ*gshT*	Spd_0150 replaced with erythromycin resistance cassette	Ref. 30
S. pneumoniae D39 Δ*copA* Δ*gshT*	Spd_0150 replaced with erythromycin resistance cassette, spd_0635 replaced with kanamycin resistance cassette	This study

Supplementary Table 4: Primers used in the study

Primer	Sequence (5’-3’)
copA_F	ATGACAGAAATTGTGAAAGCAAGC
copA_R	TTAGTCTATTTTACTAAAATTTAACAGAAGGG
copA_X	GCTTGCTTTCACAATTTCTGTCAT
copA_Y	CCCTTCTGTTAAATTTTAGTAAAATAGACTAA
copA_janus_X2	CATTATCCATTAAAAATCAAACGGAGTCTCCACCTACTCTACAATC ATC
copA_janus_Y	GGAAAGGGGCCCAGGTCTCTAAGTAATGACTAGATTTCTTTGTTAT A
copA_flank_F2	GTTGTATGGTTGACTGGACCAGA
copA_flank_R	GTGATTGCAATCCTTCTTCTTCC
copA_seq_F	CGAGTGGAAGTCATGGGAGG
copA_seq_R	CGAGTGGAAGTCATGGGAGG

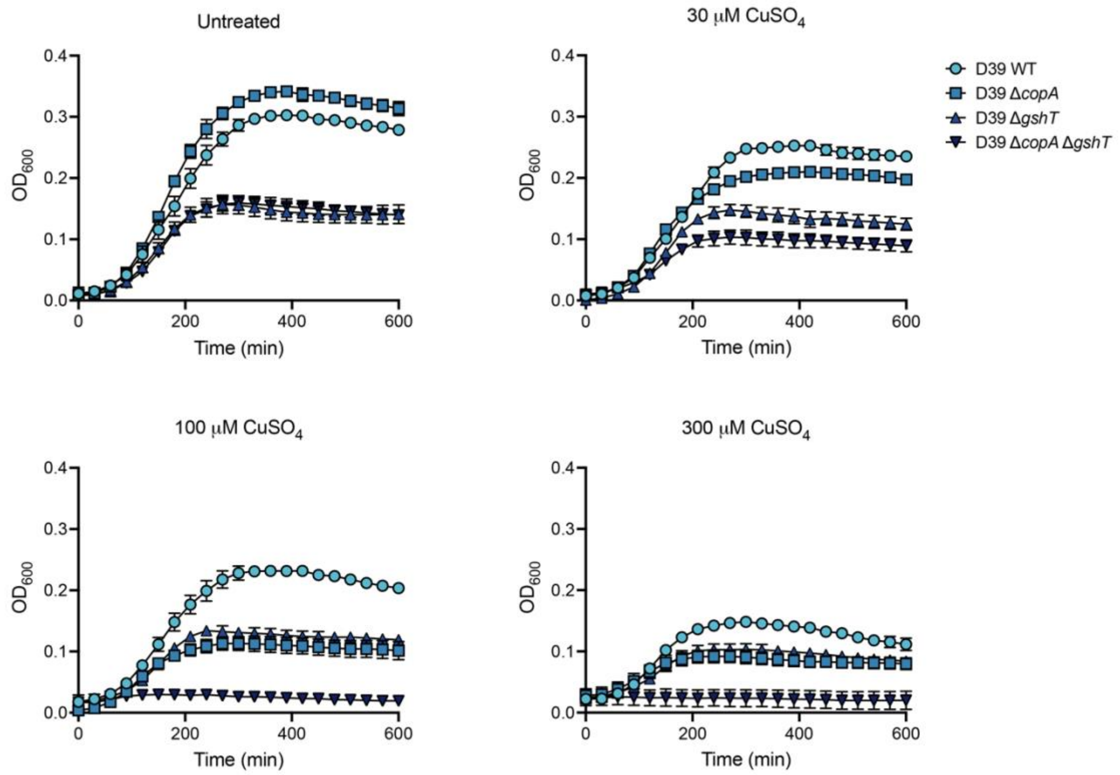

Supplementary Figure 1: Growth kinetic analyses of *S. pneumoniae* strains during Cu stress. Growth phenotypes of *S. pneumoniae* D39 (wild-type; WT) and the mutant derivative strains Δ*copA*, Δ*gshT* and Δ*copA* Δ*gshT* in the presence of $CuSO_4$ supplementation (0 µM - 300 µM). Data are representative mean (± SEM) optical density at 600 nm (OD_{600}) measurements from three independent biological experiments. Where not visible, the error bars are covered by the respective symbols.

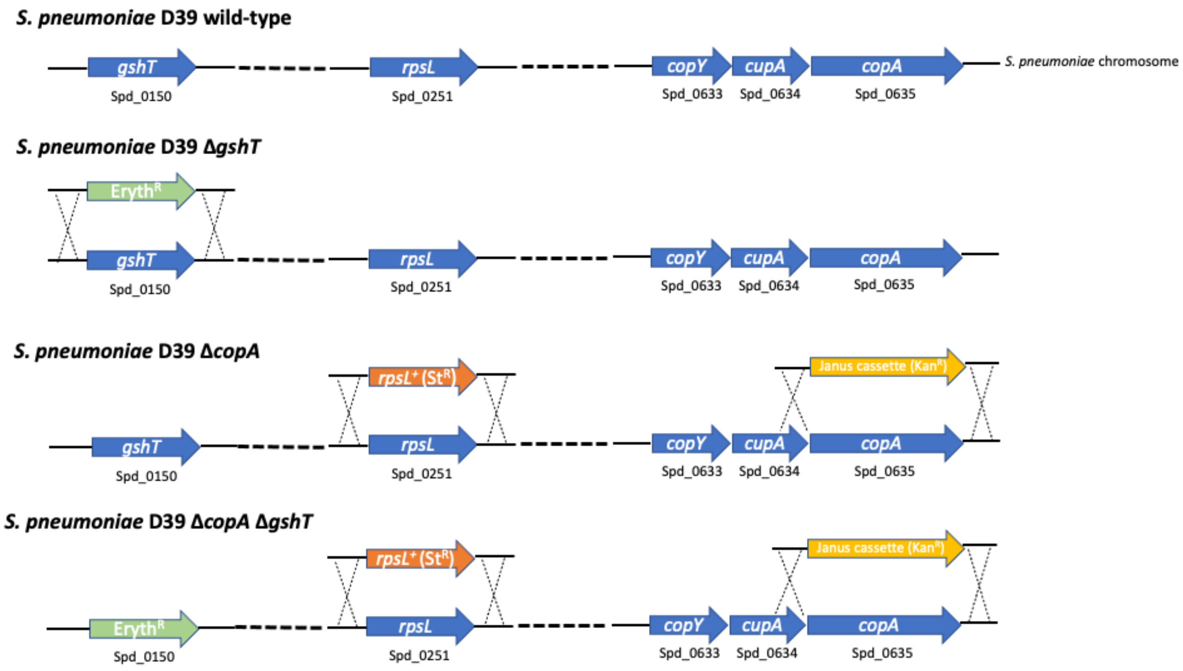

Supplementary Figure 2: Genetic organisation and mutagenesis strategy of *S. pneumoniae* wild-type, Δ*gshT*, Δ*copA* and Δ*copA* Δ*gshT*. *S. pneumoniae* wild-type genetic organisation is presented with genes of interest (blue arrows) displayed by locus tag (5'-3'). Dashed lines represent undefined distance between the annotated genes. D39 Δ*gshT* was generated in a previous study [30] via insertion of an erythromycin resistance cassette (Eryth^R; green arrow) in place of *gshT* via homologous recombination. For generation of the Δ*copA* and Δ*copA* Δ*gshT* strains, the 30S ribosomal subunit protein S12 (*rpsL*) was first replaced with a streptomycin resistant allele, *rpsL*$^+$ (orange arrow), in the D39 wild-type and Δ*gshT* genetic backgrounds, respectively. The *copA* gene was then replaced via homologous recombination with the Janus cassette [65] (yellow arrow), conferring streptomycin sensitivity and kanamycin resistance. Regions of homologous recombination have been depicted as cross-over events in dotted lines. Genetic elements are not drawn to scale.
